# Longitudinal Relations Between Parental Strain, Parent–Child Relationship Quality, and Child Well-Being During the Unfolding COVID-19 Pandemic

**DOI:** 10.1007/s10578-021-01232-4

**Published:** 2021-08-23

**Authors:** Samuel Essler, Natalie Christner, Markus Paulus

**Affiliations:** Developmental Psychology, Ludwig-Maximilians-Universität München, Leopoldstr. 13, 80802 Munich, Germany

**Keywords:** COVID-19, Parent–child relationship quality, Parental strain, Child well-being, Child problem behavior

## Abstract

As COVID-19 sweeps across the globe, scientists have identified children and families as possibly particularily vulnerable populations. The present study employed a developmental framework with two measurement points (the first at the peak of the lockdown restrictions (*N* = 2,921), the second after restrictions had been majorly loosened (*N* = 890)) to provide unique insights into the relations between parental strain, child well-being, and child problem behavior. Cross-lagged panel analyses revealed longitudinal effects of child well-being and problem behavior at T1 on parental strain at T2 with parent–child relationship quality as a moderator. True intraindividual change models showed that decreases in parental strain between measurement points predicted increases in child well-being and decreases in child problem behavior. Thus, the present research points to parental stress coping and child emotional adjustment as promising avenues for professionals and policy makers in their efforts to ensure child and family well-being throughout the pandemic.

## Introduction

The unprecedented spread of COVID-19 across the globe has had a previously unimaginable impact on human social life in virtually every country and society. From the dramatic changes of human social interactions on the micro-level to economic turmoil on the macro-level, there is almost no societal subsystem unaffected by the COVID-19 pandemic. As governments across the world have taken drastic public health measures to contain the spread of the virus, different demographic groups have faced different degrees of COVID-19 related challenges. Many politicians, professionals, and scientists have identified children and families to be among the arguably most heavily affected groups [e.g., 1, 2]. As many parents had to reorganize their work processes and switch into home offices while public education for children of all ages came to a sudden standstill in many countries, families and children could be considered one of the melting pots of the unfolding pandemic. Given the volatility of COVID-19 related societal developments and measures (e.g., closure and reopening of educational facilities, changing public health strategies based on current infection numbers), it seems paramount to investigate how the impact of COVID-19 pandemic policies on children and families changes across the span of the pandemic [e.g., 3]. Thus, the aim of the current study was to uncover the dynamics in children’s and families’ well-being and challenges during the pandemic. To this end, the present study investigated changes in children’s well-being and problem behavior during the pandemic and their relation to parental strain. One key question concerns to which extent potential relations between child well-being and parental strain are moderated by the general quality of the parent–child relationship. Importantly, the current study employed a longitudinal approach. Given that most research to date exploring the impact of the COVID-19 pandemic on children and families relied on cross-sectional designs [e.g., 2, 4, 5; for an exception see 6], little is known about the dynamic changes in the relations between child well-being and parental strain between different phases of the pandemic. Our study aimed to close this research gap.

Notably, developmental theorizing suggests that the public health-related measures taken by governments to slow the spread of COVID-19 will have a pronounced impact on children’s well-being. Clarifying this impact, understanding aggravating and mitigating factors, and learning about potential relations to child and parental social functioning will give crucial information in ensuring child well-being in the further phases of the pandemic to come. From a bioecological perspective [7], child well-being and adjustment relies on proximal as well as distal social factors [see also 8]. As the COVID-19 pandemic heavily impacted distal (e.g., social disruptions such as the far-reaching lockdown of social life) as well as proximal (e.g., drastic reduction of social interactions with peers, teachers, or grandparents during the lockdown) layers, one would assume negative effects on children’s emotional well-being. This prediction is especially underscored by research showing that children’s interactions with social agents beyond the core family constitutes an important resilience factor [9]. In addition, children, especially during early and middle childhood, depend heavily on their caregivers due to their emerging but yet limited cognitive [e.g., 10], self-regulative [e.g., 11], and social-emotional [e.g., 12] capacities. That is, children might not fully understand the pandemic as the cause of the dramatic changes in their lives, they might experience strong emotional reactions such as anxiety concerning their own possible infection and that of close others, and they might be in exceptional need for (external) self-regulatory resources in order to navigate the disruptive situation at hand. First evidence shows that especially during the initial phase of the pandemic, parents reported children’s disturbed sleep patterns, more-challenging bedtime routines, and children’s decreased sleep quality [6]. A recent review on the COVID-19 pandemic’s impact on the mental health of children and adolescents underscores this point by reporting high rates of anxiety, depression, and post-traumatic symptoms among children [13]. Thus, understanding the impact of COVID-19 related policies directly speaks to the relevance of a bioecological perspective on child development. Taken together, it is important to investigate the developmental dynamics of children’s well-being during the ongoing COVID-19 pandemic. To assess the unfolding developmental dynamics, the present study employs a wide range of indicators of child well-being and problem behavior at two measurement points (the first at the peak of the lockdown restrictions, the second after restrictions had been majorly loosened) during the pandemic.

Influential developmental theories on child-caregiver interactions [e.g., 13, 14; for reviews see 15, 16] have proposed that especially young children require the availability of their caregivers as well as sensitive caregiving during challenging phases [e.g., 17, 18]. That is, as children might experience negative arousal (e.g., insecurity, anxiety, disorientation) during social upheaval caused by the COVID-19 pandemic, they rely on their caregivers to help them regulate their emotions, to support them in understanding the current situation according to their cognitive capacities, and to anticipate and react adequately to their needs [e.g., 13, 14, 19]. It is well established that sensitive and responsive caregiving is related to children’s socio-emotional development and self-regulation skills [e.g., 20–23]. In the context of the COVID-19 pandemic, this leads to the paradoxical situation that especially while children’s need for caregiver availability increases during the COVID-19 crisis, caregivers themselves are also experiencing unprecedented strain. That is, many caregivers are under intense pressure to manage new working situations (e.g., home office, temporary leave, reduced working hours) as well as cope with increased caregiving time due to the closure of educational facilities [4]. In addition, a substantial percentage of caregivers reported a decrease of household income and/or an increase in household debt [5]. In somehow comparable contexts, caregivers’ warmth decreased and harsh parenting increased [e.g., during the 2008 global recession: 24–26]. From a developmental perspective, parental strain is known to be detrimental to child development. A large set of empirical studies has demonstrated negative effects of parental strain on children’s physical, social-emotional, and cognitive development [e.g., 27, 28]. For example, one study found longitudinal reciprocal effects between parenting stress and child externalizing behavior for children aged 4 to 10 years [29]. Thus, theoretical considerations point to parental stress as an important construct to address when investigating the impact of COVID-19 related pandemic policies on children and families [e.g., 30]. As parental stress in response to COVID-19 related policies can be subject to quick changes as the COVID-19 environment (e.g., restrictions, working conditions, operation of educational facilities) evolves, it constitutes a variable expected to be volatile over the course of the pandemic. Following the above theoretical considerations, these changes in parental stress manifest themselves at the level of caregiver-child interactions (e.g., sensitive and responsive caregiving), which in turn might impact children’s well-being. Subsequently, two major questions concerning the longitudinal role of parental strain arise. First, are there specific longitudinal relations between parental strain and child well-being and problem behavior, possibly even reciprocal, during the COVID-19 pandemic? Second, is situation-related parental change in stress related to the situation-related change in children’s well-being and problem behavior? That means, do the dynamics of change in parental well-being affect the dynamics of change in child well-being across the pandemic-related changing context? Importantly, large scale studies during the pandemic such as the current one present an unique opportunity since the dawn of empirical developmental research to test, amongst others, theories relating caregiver qualities to child well-being. The current study aims to address this question in detail.

Besides the volatile parental strain construct, developmental theories also point to the important role of more stable and enduring concepts during the COVID-19 pandemic such as the parent–child relationship quality and parental self-efficacy in child-rearing. That is, other than parental strain, which is directly affected by macrostructural changes induced by the COVID-19 pandemic, the parent–child relationship quality describes the general nature of the relationship prior to and less affected by the COVID-19 pandemic. First evidence seems to underscore this proposition as the majority of parents does not report a change in the parent–child relationship quality during the peak of the pandemic [5]. Likewise, parental self-efficacy can be conceptualized as parents’ general conviction of their child rearing competencies regardless of the COVID-19 pandemic. Thus, both can be viewed as a possible risk or protective factors in the adjustment to COVID-19 pandemic policies.

Turning to relationship quality first, the many uncertainties around the COVID-19 pandemic and children’s dependency on their caregivers constitute strong theoretical reasons to view it as a pivotal factor accounting for children’s positive adjustment in times of the pandemic [1, 31; see also 32]. A vast amount of literature points to the relations between parent–child relationship quality and a number of child and adolescent outcomes [e.g., 33–35]. For example, one study showed that a positive relationship quality in middle childhood and adolescence can act as a buffer by mitigating the adverse effects of peer stressors on symptoms of depression [33]. Specifically, one could conceive of children being better able to cope with periods of higher parental stress if they can rely on a positive relationship to their parents in general. Thus, the parent–child relationship quality can be conceptualized as a moderator of the effect of parental stressors on child well-being. From a theoretical perspective, a positive parent–child relationship quality might be an important protective factor during the COVID-19 pandemic. That is, parent–child relationship quality might moderate effects of parental strain on child well-being in such a way that a positive relationship will buffer negative effects of parental strain. In other words, the parent–child relationship quality might change the strength of the effect between parental strain and child well-being in such a way that the more positive the parent–child relationship, the weaker the effect of parental strain on child well-being.

A further theoretically relevant parental quality in the context of the COVID-19 pandemic is parents’ belief in their ability to perform their parenting role successfully (i.e., parental self-efficacy concerning child-rearing; [36]). Numerous studies underscore the positive effects of parental self-efficacy on child psychological functioning and assessment [e.g., 37–39; for a review see 40]. For example, one study found that parental self-efficacy was negatively related to anxiety as reported by preschoolers and that parental self-efficacy could act as protective factor in the development of anxiety [38]. Theoretically speaking, high values of parental self-efficacy could have positive effects on child well-being and problem behavior especially during the intense COVID-19 related lockdown restrictions, as parenting roles faced the arguably biggest challenges during this time frame.

## The Current Study

The present study employs a longitudinal developmental framework to investigate temporal dynamics of child well-being and problem behavior during the early phase of the COVID-19 pandemic. Thus, the current study aims at deepening our knowledge of longitudinal effects of COVID-19 pandemic policies on children’s well-being. Thereby, the present research complements a previous study reporting cross-sectional findings from the first lockdown period (T1) within the same sample [30]. Specifically, the present research advances developmental theorizing by investigating if and to what extent children’s well-being and problem behavior change alongside the changes in distal factors (e.g., loosening of public health-related lockdown restrictions from first to second measurement point). That is, the current study tests propositions of bioecological models stressing the importance of the macrostructural environment and its changes for child development. In addition, it investigates theoretical claims regarding the importance of caregiver availability and caregiver stress for children’s well-being in challenging situations. Finally, it examines theoretical accounts suggesting that the parent–child relationship quality is a crucial protective factor in children’s adjustment to the pandemic. Taken together, the current study assesses a number of theoretical developmental aspects relevant to risk factors and psychopathological conditions during the pandemic and beyond. That is, by investigating the impact of the COVID-19 pandemic on child well-being in behavioral and emotional domains, the present study aims to contribute to identifying crucial developmental changes that might bear on clinical conditions and psychopathological development in children over the course of the pandemic.

In the present study, parents reported on child problem behavior and well-being as well as parental strain, parental self-efficacy, and parent–child relationship quality. Given the above theoretical consideration concerning especially young children’s proposed vulnerability to the social disruptions during the COVID-19 pandemic, we focused on 3 to 10 year old children.

Based on the theoretical propositions above, we predicted that (1) cross-sectionally within T1, parental self-efficacy, parent–child relationship quality, and parental stress would relate to child well-being and problem behavior, that (2) parental stress would decrease and child well-being would increase during the transition of strict restrictions (T1) to loosened restrictions (T2), that (3) parental strain at T1 would negatively predict child well-being and positively predict problem behavior at T2, that (4) decreases in parental strain between T1 and T2 would predict increases in child well-being and decreases in problem behavior between T1 and T2, that (5) as a protective factor the parent–child relationship quality would moderate the effects in hypotheses three and four in such a way that the more positive the parent–child realationship quality the weaker the adverse effects of parental strain on child well-being and problem behavior.

## Method

### Participants

At the first measurement point (T1), the final sample consisted of 2921 participants. We excluded 283 additional participants who started the questionnaire, but did not proceed with answering questions pertaining to the key variables (parental strain, child well-being and problem behavior, parental self-efficacy, relationship quality *n* = 183) and participants who reported no valid age of the child or an age outside the age range of 3–10 years (*n* = 100). Out of the 2921 participants at T1, 1851 participants gave their consent and provided their e-mail address to be invited to a follow-up questionnaire (i.e., T2). At the second measurement point (T2), a total of 890 participants (out of 1,851 invited participants) answered the follow-up questionnaire (retention rate of 48%) and made up the final T2 sample. We excluded an additional 324 participants due to incomplete answers (*n* = 99), due to children’s ages missing or outside the 3–11 years age range (*n* = 16), due to the failure to match participants’ answers at T1 and T2 based on the ID-codes (*n* = 157), and due to non-matching age and gender variables between T1 and T2 (*n* = 52). Importantly, we collected data for T1 at the pinnacle of the health-related lockdown restrictions in Germany (end of April—beginning of May 2020) to cover the arguably most challenging phase of the COVID-19 pandemic for children and families so far. During this time frame, educational facilities were closed down with digital forms of learning yet to be installed (e.g., virtual schools). Kindergartens were open only on an emergency care schedule for a small subgroup of children whose parents pursued highly system-relevant professions (e.g., physicians). In addition, government policies restricted physical social interactions to one’s own household and implemented nationwide curfews. Data for the second measurement point, to which families who had already participated at T1 were openly invited, was collected in the middle of July 2020 when the major lockdown restrictions (e.g., meeting people from other households) had been loosened. Participants were recruited via online postings, Email invitations to families associated with the lab, and by words of mouth.

The demographic characteristics of the sample are displayed in Table 1. The local ethics committee approved the study as part of a larger longitudinal project on the impact of the COVID-19 pandemic on children and families. Participants provided their informed consent and could take part in a raffle of ten (T1) and five (T2) 50 € gift vouchers at the end of the questionnaire.

**Table 1:** Key demographic characteristics of the sample at T1 (N = 2921) and T2 (N = 890)

Demographic variable	T1	T2
*Vocational degree*		
University degree	49%	–
Vocational training	24%	–
University of applied sciences degree	15%	–
Professional academy	8%	–
Master training	3%	–
No vocational degree	1%	–
*Current job status*		
Home office	44%	31%
Job outside of the home	18%	35%
Parental leave	17%	18%
Reduced working hours	6%	4%
No job	5%	5%
Exempted	4%	2%
Other	6%	5%
*Change in attendance of educational institutions*		
Yes, my child visits preschool again	–	52%
Yes, my child visits school again	–	34%
No, my child continues to visit an institution	–	6%
Yes, my child visits a daycare center again	–	4%
No, my child continues to visit no institution due to COVID-19	–	3%
No, my child continues to visit no institution	–	1%
*Change in further extra familiar childcare (grandparents, nanny, …)*		
No, my child continues to receive no extra familial childcare	–	40%
Yes, my child receives extra familial childcare again	–	34%
No, my child continues to receive no extra familial childcare due to COVID-19	–	18%
No, my child continues to receive extra familial childcare	–	7%

### Power Analysis

Using the R-package semPower [41], we conducted a statistical power analysis for SEM models. Specifying the effect size as RMSEA = 0.05, alpha = 0.05, power = 0.80, and 10 degrees of freedom, the required sample size was 651. In addition, we followed Kline’s [42] guideline of more than 100 participants for a medium sample size for SEM analyses. Therefore, we aimed at a final sample of at least *N* = 700.

### Materials

As this study aimed at uncovering longitudinal parent and child dynamics during the COVID-19 pandemic, we primarily focus on the measures relevant for investigating change between T1 and T2. For both measurement points, the online survey comprised three blocks: (1) demographics and parental strain, (2) situation of the child during the COVID-19 pandemic, as well as (3) general measures of parental self-efficacy and parent–child relationship quality. In the introduction, we asked participants to have the primary caregiver of both parents in terms of time complete the survey at both measurement points. In the following, we present the three blocks consecutively. Given that the survey at T2 was largely a shortened version of the T1 survey, we describe the T1 survey below and indicate the parts that were also included in the T2 survey.

The rationale for assessing some variables at both measurement points and some variables only at T1 were theoretical considerations about which variables would be expected to change to a notable degree between the two measurement points. That is, especially parental strain, child well-being, and child problem behavior were expected to change to a noticeable extent over the course of the pandemic, whereas parent–child relationship quality, parental self-efficacy concerning child rearing, and parental strategies were expected to remain more stable.

#### Demographics

The first block consisted of demographic questions concerning the parent and the child. Regarding the parent, participants indicated their age (T1 and T2), gender (T1 and T2), family and partner status (only T1), gender of partner (only T1), number of children in the household (only T1), state of residence (only T1), housing situation (only T1), educational degree of self and partner (only T1), current job status of self and partner (T1 and T2), and relative childcare work of both partners in percent. Moreover, participants answered how many more hours they spent caring for the child on a daily basis as compared to before the pandemic (T1 and T2). Concerning the child, demographic questions assessed age (T1 and T2), gender (T1 and T2), and educational institution (kindergarten, school).

#### Parental Strain (T1 and T2)

We included a set of three questions concerning parental strain as compared to before the pandemic (“I feel more strained in the current situation than normally”, “The current situation is more challenging for me than normally”, “I feel more stressed out in the current situation than normally”; Cronbach’s α (T1) = 0.91, Cronbach’s α (T2) = 0.94). Parents answered on a Likert scale ranging from 1 (“do not agree at all”) to 5 (“totally agree”). The scoring of parental strain relied on these same three items for both measurement points. For the subsequent analyses, we computed the mean across the three items to form the *parental strain* variable. In addition, we assessed only at T2 how the extra familiar child care situation changed between T1 and T2. That is, one question assessed possible changes in children’s attendance of educational institutions. A second question assessed possible changes in further extra familiar childcare arrangements (e.g., grandparents did not look after the child at T1 due to COVID-19 lockdown restriction but grandparents looked after the child again at T2).

#### Situation of the Child During the COVID-19 Pandemic

##### Child Well-Being: KIDSCREEN (T1 and T2)

To measure the impact of the COVID-19 pandemic on the child, we modified 12 items from the German translation of the 52-items KIDSCREEN Health-Related Quality of Life Questionnaire for Children and Adolescents (Ravens-Sieberer et al., 2006). The KIDSCREEN-52 was designed to measure 10 dimensions of health and well-being (physical and psychological well-being, moods and emotions, self-perception, autonomy, parent relations and home life, social support and peers, school environment, social acceptance, and financial resources) in healthy and chronically ill children and adolescents. Cronbach’s Alphas for the dimensions range between 0.76 and 0.89. The KIDSCREEN evidences good convergent and discriminant validity [43].

There were three main reasons for using a specific selection of items (see below for wording of the items used). First, COVID-19 related lockdown restrictions and social distancing measures made some scales inapplicable (e.g., friends, school and learning, others). That is, as there were practically no social interactions or digital educational setups during T1, we dropped items relating to interactions between friends and items relating to the school/kindergarten setting. Second, the wording for a couple of items was very similar and given the time constraints of the survey we only used one of these items (e.g., “was in a good mood” but not “was happy”; “enjoyed life” but not “was satisfied with life”). Third, we selected scales (e.g., “feelings” and “general mood” but not “physical activities” and “health”) based on theoretical relevance for assessing the impact of the COVID-19 pandemic on children and families. In addition, we modified the items to not assess quality of life at a single time point (as in the original version), but to assess positive and negative changes in quality of life between the time proceeding the COVID-19 pandemic and the time of the strictest lockdown measures (T1) as well as the time of the loosened lockdown restrictions (T2). That is, participants rated on a 7-point scale how much more or how much less their child had positive emotions, moods, time for itself and with its parents (four subscales in total) during the weeks of the complete lockdown (T1) and during the weeks of the loosened lockdown restrictions (T2) as compared to before the COVID-19 pandemic. The response scale ranged from 1 (“clearly less”) to 7 (“clearly more”) with the middle category 4 denoting “no difference”.The items were (“Compared to the situation before the COVID-19 pandemic, my child (item 1–12) in the last weeks?”): (1) enjoyed life, (2) was in a good mood, (3) had fun (1–3 aggregated to scale “emotions”; Cronbach’s α (T1) = 0.88, Cronbach’s α (T2) = 0.91), (4) was sad, (5) felt so bad that s/he did not want to do anything, (6) was lonely (4–6 aggregated to scale “moods”; Cronbach’s α (T1) = 0.78, Cronbach’s α (T2) = 0.83), (7) was content (single item scale for “life satisfaction”), (8) had time for himself/herself, (9) was able to do things s/he wanted to do in its free time (8–9 aggregated to scale “free time”; Cronbach’s α (T1) = 0.40, Cronbach’s α (T2) = 0.28—as this subscale failed to reach acceptable reliability values, it was not used for our analyses), (10) felt that its parents had time for it, (11) felt fairly treated by its parents, and (12) has been able to talk to its parents when s/he wanted (10–12 aggregated to scale “family”; Cronbach’s α (T1) = 0.71, Cronbach’s α (T2) = 0.72). For the subsequent analyses, we first recoded the three reversely coded items of the moods subscale. Then, we computed the mean across items for all subscales except free time (due to the low reliability). The three subscales of children’s well-being that all addressed children’s emotional well-being (emotions, moods, life satisfaction) were highly interrelated at T1 (*r*s > 0.69) and at T2 (*r*s > 0.76), so we calculated the mean across emotions, moods, and life satisfaction (referred to as *emotional well-being* in the subsequent analyses). The subscale “family” entered the following analyses as *family-related well-being.*

##### Child Problem Behaviors (T1 and T2)

To assess the child’s behavioral and emotional problems at both measurement points, we modified the subscales (emotional symptoms, conduct problems, hyperactivity-inattention) and items of the Strengths and Difficulties Questionnaire [SDQ; 44]. Reliabilities of the original SDQ subscales evidenced acceptable to good values and range between Cronbach’s α = 0.58–0.76 [45]. Each subscale consists of 5 items, the language of administration was German. Parents were asked to report on their child’s problem behavior with respect to the last three weeks. Thus, we ensured that the reported time frame was located completely within the lockdown period (for T1) and completely within the period of loosened restrictions (for T2). For the same reason as above with the KIDSCREEN, we chose these three subscales as further subscales largely focused on social interactions with other children. Given the COVID-19 related social distancing measures (e.g., closure of educational facilities, strict policies prohibiting social interactions between households), we had to modify and shorten some of the remaining items (e.g., remove references to behavior at school or towards other children). To keep the item structure similar overall and to avoid ambiguous item formulations (e.g., “Often unhappy, depressed, or tearful”), we also adapted the remaining items as follows: emotional problems (“Often complains of headaches”, “Has many worries”, “Often unhappy”, “Nervous or clingy”, “Has many fears”; Cronbach’s α (T1) = 0.77, Cronbach’s α (T2) = 0.75), conduct problems (“Often has temper tantrums”, “Generally obedient”, “Often fights”, “Often lies or cheats”, “Steals from home”; Cronbach’s α (T1) = 0.71, Cronbach’s α (T2) = 0.69), and hyperactivity (“Restless, overactive”, “Constantly fidgeting”, “Easily distracted”, “Reflects”, “Sees tasks through to the end”; Cronbach’s α (T1) = 0.66, Cronbach’s α (T2) = 0.66). Participants answered on the original 3-point scale (0 – “not true, 1 – “somewhat true”, 2 – “certainly true”). After recoding the three reversely coded items, we calculated sum scores for each of the subscales. As all subscales of problem behavior (emotional, conduct, hyperactivity) were highly interrelated at T1 (*r*s > 0.35) and T2 (*r*s > 0.31), we computed the mean across the three subscales as an overall measure of children’s problem behavior (referred to as *problem behavior* in subsequent analyses). Given that mean scores and sum scores are perfectly correlated and therefore lead to the same results in regression-based statistical analyses, we used the mean score to combine the three subscales as it enhances interpretability.

#### Parental Self-efficacy (only T1) and Parent–Child Relationship Quality (T1 and T2)

The third part of the questionnaire was intended to assess more enduring and general parental and relationship qualities that are characteristic for our participants. To measure parental self-efficacy (only T1), we included an established parenting self-efficacy questionnaire (The Parenting Self-Efficacy Questionnaire—FSW), consisting of 9 items [46]. The questionnaire assesses the unidimensional construct of parenting self-efficacy (example item: “I think that I am capable of everything a mother/a father needs to be capable of.”). The original FSW showed good psychometric properties (Cronbach’s α = 0.78). Participants provided answers on a 4-point Likert scale ranging from 1 (“disagree”) to 4 (“agree”). We calculated the mean across all 9 items for the subsequent analyses. In addition, we assessed the quality of the parent–child relationship at both measurement points by adapting 8 items from the Network of Relationships Inventory [NRI; 47]. The original NRI questionnaire evidenced acceptable to good reliability values with Cronbach’s α > 0.60 for all relevant scale scores [47]. The items combined into four pairs made up the scales “Intimacy” (Cronbach’s α (T1) = 0.88, Cronbach’s α (T2) = 0.89), “Admiration” (Cronbach’s α (T1) = 0.70, Cronbach’s α (T2) = 0.71), “Conflict” (Cronbach’s α (T1) = 0.80, Cronbach’s α (T2) = 0.84), and “Dominance” (Cronbach’s α (T1) = 0.66, Cronbach’s α (T2) = 0.72; example item: “My child tells me what he/she is thinking” from the intimacy scale). The response format ranged from 1 (“never”) to 5 (“very often”). Given that the positive aspects (intimacy and admiration; T1: *r* = 0.29; T2: *r* = 0.33) and the negative aspects (conflict and dominance; T1: *r* = 0.23; T2: *r* = 0.26) correlated most strongly with each other, we further calculated one mean for the positive aspects and one comprising the negative aspects (referred to as negative and positive aspect of the relationship quality in subsequent analyses).

### Procedure

We hosted the questionnaire on Qualtrics for both measurement points. The average response time was approximately 15 min at T1 and about 7 min at T2. Introductory instructions explained the purpose of the study and informed participants about data privacy topics. All participants agreed to the anonymous storage of their data at both measurement points.

Next, participants completed the survey in a fixed order (see Materials for details). The first block consisted of the demographic questions and the items on parental strain. The second block covered the child’s behavior and well-being as well as parental strategies (only T1). Finally, the third block consisted of questions about parenting self-efficacy (only T1) and about the parent–child relationship quality.

### Analyses

All analyses were computed in R 4.0.2. We used the package lavaan for testing all models [48]. To examine longitudinal dynamics between COVID-19 related processes, we employed both Cross-Lagged Panel Models and True Intraindividual Change models. *Cross-Lagged Panel Models (CLPM)* allow to identify relations between variables across time and inform about the directionality of longitudinal relations. The basis for CLPMs are two (or more) variables which are assessed at two (or more) time points. In this case, we assessed parental strain and child problem behavior at two measurement points. Subsequently, there are three types of effects one can identify: (1) cross-sectional effects, that is, the relation between the measured variables within each measurement point (e.g., correlation between parental strain and child problem behavior at T1); (2) stability effects, that is, the temporal relations of a given variable across measurement pointes (e.g., the stability of parental strain from T1 to T2); and (3) cross-lagged relations, that is, longitudinal effects of one variable on the other (e.g., effects of parental strain at T1 on child problem behavior at T2). Typically, these cross-relations are the focal point of interest as they allow to investigate longitudinal effects between variables. *True Intraindividual Change (TIC) Models* are adapted path models that allow to test predictors of intraindividual change between two measurement points [49]. For that purpose, the variables of interest are modeled as state and change variable. In particular, measurements from T1 were defined as baseline variables and, following previous developmental research [cf. 23], latent change variables were computed to model intraindividual change. In detail, a latent baseline variable predicted the variable of interest at T1 and T2 and a latent change variable predicted the variable of interest only at T2.

We included all participants who completed at least the first key variable (parental strain). Missing data on the other key variables is as follows: At T1, data on child well-being is missing for 3% of participants, on child problem behavior for 4% of participants, and for relationship quality for 9% of participants. At T2, data on child well-being is missing for 1% of participants, on child problem behavior for 2% of participants, and for relationship quality for 2% of participants. Following Little’s MCAR test, missing data on the key variables can be considered as missing completely at random both at T1 (*χ*^*2*^ = 44.61, *df* = 35, *p* = 0.128) and T2 (*χ*^*2*^ = 31.82, *df* = 35, *p* = 0.622). Following Graham [50], we used full information maximum likelihood estimation in our longitudinal analyses to account for missing data.

**Fig. 1:**
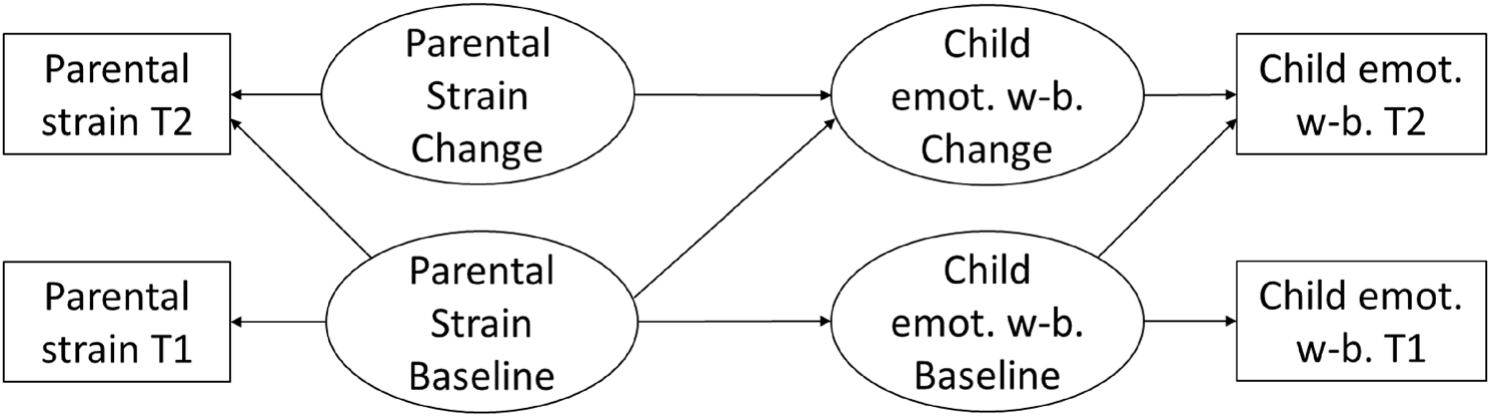
Representation of TIC models, exemplary for children’s emotions as one aspect of well-being. Boxes represent manifest variables, circles represent latent variables

In order to evaluate model fits, we relied on χ^2^ difference tests, Root Mean Square Error of Approximation (RMSEA), Standardized Root Mean Square Residual (SRMR), and Comparative Fit Index (CF). Note, the χ^2^ test is significant for most of our models although other fit parameters indicate acceptable or good model fit. The significant χ^2^ test might result from the large sample size rather than indicate insufficient model fit [51]. Data supporting our analyses are openly available on OSF at https://osf.io/7dn3y/ (Fig. 1).

## Results

For descriptive purposes, Table 2 presents means and standard deviations of key variables at the two measurement points for the final longitudinal sample. A zero-order correlation matrix of these variables is displayed in Table 3. In order to examine differences of the means between T1 and T2, we computed paired-sample t-test (see Table 2). Parental stress and children’s problem behavior decreased from T1 to T2. Children’s emotional well-being increased while family-related well-being decreased. Positive aspects of the parent–child relationship quality slightly decreased across time.

**Table 2:** Means and standard deviations for key variables at T1 and T2 for the final sample at T2

	T1		T2		Mean comparison		
Variable	*M*	*SD*	*M*	*SD*	*t*	*df*	*p*
Parental stress	4.01	1.03	3.24	1.18	18.69	889	< .001
Well-being: emotional	3.40	1.17	4.29	1.12	− 17.66	876	< .001
Well-being: family	4.21	1.10	4.04	0.85	4.34	876	< .001
Problem behavior	3.47	1.85	2.86	1.63	12.74	874	< .001
Relationship quality: positive	4.29	0.53	4.14	0.52	11.03	873	< .001
Relationship quality: negative	2.76	0.49	2.80	0.51	− 3.05	873	.002
Parental self-efficacy	3.13	0.40	–	–	–	–	–

Comparison of the means using paired-sample t-tests

**Table 3:** Zero-order Pearson correlations of key variables at T1 and T2 for the final sample at T2 (N = 890)

Variable	1	2	3	4	5	6	7	8	9	10	11	12
*T1*												
1. Parental stress												
2. Well-being: emot	− .54***											
3. Well-being: family	− .41***	.51***										
4. Problem behavior	.51***	− .58***	− .36***									
5. RQ: positive	− .06^+^	.02	.05	− .19***								
6. RQ: negative	.12***	− .10**	− .02	.29***	− .14***							
7. Parental self-effic	− .14***	.14***	.11**	− .32***	.44***	− .41***						
*T2*												
8. Parental stress	.40***	− .31***	− .21***	.33***	− .05	.06^+^	− .09**					
9. Well-being: emot	− .07*	.14***	.13***	− .09*	.02	− .04	.02	− .22***				
10. Well-being family	− .09*	.12***	.23***	− .09*	.04	− .04	.06	− .22***	.38***			
11. Problem behavior	.30***	− .33***	− .21***	.68***	− .21***	.29***	− .32***	.37***	− .32***	− .22***		
12. RQ: positive	− .09**	.11**	.14***	− .18***	.67***	− .13***	.39***	− .11**	.09*	.16***	− .26***	
13. RQ negative	.16***	− .12***	− .03	.33***	− .15***	.63***	− .37***	.18***	− .11**	− .07*	.46***	− .23***

*RQ* parent–child relationship quality

^+^*p* < .01, *p < .05, **p < .01, ***p < .001

### Cross-Sectional Analyses for T1 (*N* = 2921)

In order to examine influencing factors of children’s well-being and problem behavior at the peak of COVID-19 related restrictions (within T1), we computed multiple linear regressions. One regression focused on children’s emotional well-being as outcome variable, one focused on children’s family-related well-being, and one focused on children’s problem behavior. We included parental strain, positive and negative aspects of parent–child relationship quality, and parental self-efficacy as predictors. Predictors were entered simultaneously into each regression model (i.e., we ran three separate regression models with the same predictors). Results are presented in Table 4. Most important, parental strain was significantly associated with children’s well-being, both emotional and family-related. The higher parental strain, the lower children’s well-being was reported. Problem behavior was most strongly associated with parental strain. In addition, relationship quality and parental self-efficacy were significantly related to problem behavior. The higher parental strain, the higher negative aspects of relationship quality, the lower positive aspects of relationship quality, and the lower parental self-efficacy, the higher child problem behavior was reported.

**Table 4:** Multiple linear regressions on children’s emotional well-being, family-related well-being and problem behavior within T1

	Emotional well-being			Family-related well-being			Problem behavior		
	*β*	*p*	95% CI	*β*	*p*	95% CI	*β*	*p*	95% CI
Parental strain	− .49	< .001	[− .59, − .52]	− .40	< .001	[− .47, − .40]	.43	< .001	[.72, .84]
RQ (positive)	− .01	.450	[− .11, .05]	.03	.137	[− .02, .14]	− .06	< .001	[− .34, − .09]
RQ (negative)	.01	.581	[− .06, .11]	.01	.678	[− .07, .10]	.15	< .001	[.44, .70]
Parental s.-eff	.03	.133	[− .03, .21]	.04	.055	[− .00, .23]	− .14	< .001	[− .86, − .50]
*R*^*2*^*, p*	.25	< .001		.17	< .001		.31	< .001	

Standardized regression coefficient, p-value, and 95% confidence interval for each predictor

*RQ* parent–child relationship quality

### Cross-Lagged Panel Models Across T1 and T2 (*N* = 890)

**Fig. 2:**
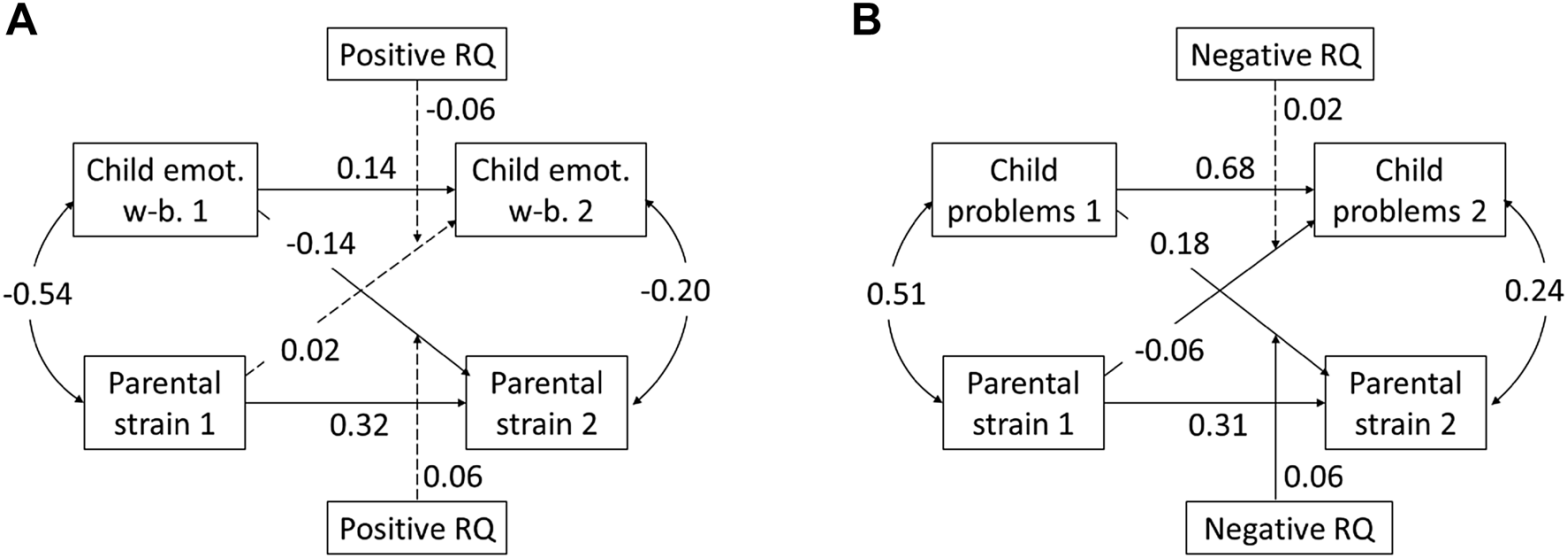
Cross-lagged panel models regarding parental strain and children’s emotional well-being (**A**) and regarding parental strain and children’s problem behavior (**B**) with positive/negative aspect of parent–child relationship quality (RQ) as moderator. Dashed arrows: n.s.; continuous arrows: p < .05

We computed separate CLPMs on mean-centered variables for the three aspects of children’s well-being (emotional, family) and problem behavior to investigate stabilities and cross-relations between parental strain and the respective child variable. In order to examine whether the cross-relations between parental strain and child behavior depend on the parent–child relationship quality, we included the interaction terms between relationship quality at T1 and the respective T1 variables in each model. We included both interaction terms for the cross-relation from child variable to parental strain and vice versa. To address both positive and negative aspects of relationship quality, we computed separate models for the two aspects. Thus, for each child variable (emotional well-being, family-related well-being, problem behavior), we computed two CLPMs, one addressing the moderating effect of the positive aspect of relationship quality and one addressing the negative aspect of relationship quality. Figure 2 displays an exemplary model regarding emotional well-being and problem behavior.

#### Parental Strain and Children’s Well-Being

All models addressing children’s well-being (emotional aspect; family) revealed an acceptable model fit, with χ^2^ (8, n = 890) < 32.6, *p* < 0.071, RMSEA < 0.06, SRMR < 0.03, CFI > 0.95. The two models on children’s *emotional well-being (emotions, moods, life satisfaction)*, addressing the moderating effect of positive and negative aspects of relationship quality, revealed stability of parental strain, *β*s > 0.31, *p*s < 0.001, and of children’s emotional well-being, *β*s > 0.14, *p*s < 0.001, from T1 to T2. Within each measurement point, parental strain and children’s emotional well-being were negatively related, T1: *β* = − 0.54, *p* < 0.001; T2: *β* = − 0.20, *p* < 0.001.

Concerning relations across time points, the models revealed significant negative cross-relations from children’s emotional well-being to parental strain, *β*s < − 0.13, *p*s < 0.001, but not vice versa, *β*s < 0.02, *p*s > 0.681. That means the worse children’s well-being at T1, the higher parental strain at T2.The cross-relation from children’s emotional well-being to parental strain tended to be moderated by the positive aspect of parent–child relationship quality, *β* = 0.06, SE = 0.06, *p* = 0.065: Children’s emotional well-being at T1 was negatively related to parental strain at T2, particularly if the positive aspect of parent–child relationship quality at T1 was low. The moderation of the cross-relation from parental strain to children’s emotional well-being was also not significant, but approached the level of significance, *β* = − 0.06, SE = 0.07, *p* = 0.056. All interactions with negative aspects of parent–child relationship quality were non-significant, *β*s > − 0.04, *p*s > 0.243.

The two models on children’s *family-related well-being* revealed stability of parental strain, *β*s = 0.37, *p*s < 0.001, and of children’s well-being, *β*s = 0.23, *p*s < 0.001. Within each measurement point, parental strain and children’s family-related well-being were negatively related, T1: *β*s = -0.41, *p* < 0.001; T2: *β* = -0.19, *p* < 0.001.

Concerning relations across time points, the models revealed no significant cross-relations, neither from parental strain to children’s family-related well-being, *β*s < 0.02, *p*s > 0.672, nor vice versa, *β*s = − 0.06, *p*s > 0.073. Likewise, parental strain did not interact with any aspect of relationship quality in predicting children’s family-related well-being, *β*s < 0.04, *p*s > 0.190.

#### Parental Strain and Children’s Problem Behavior

The models revealed an acceptable model fit, χ^2^ (8, n = 890) < 53.2, *p* < 0.001, RMSEA < 0.08, SRMR < 0.04, CFI > 0.96. Both models, one addressing the moderating effect of positive aspects and one addressing negative aspects of relationship quality, revealed stability of parental strain, *β*s = 0.31, *p* < 0.001, and children’s problem behavior, *β*s > 0.68, *p* < 0.001, from T1 to T2. Within each measurement point, parental strain and children’s problem behavior was positively related, T1: *β* = 0.51, *p* < 0.001; T2: *β* = 0.24, *p* < 0.001.

**Fig. 3:**
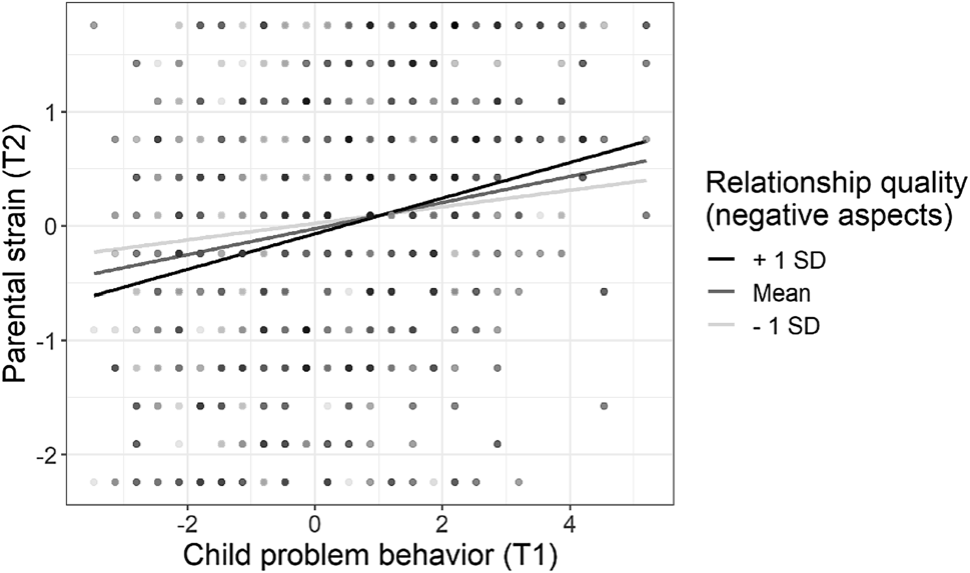
Interaction between child problem behavior at T1 and negative aspects of relationship quality on parental strain at T2 (mean-centered scores). Slopes are depicted for low (− 1 SD), medium (mean), and high (+ 1 SD) levels of negative relationship quality aspects

From T1 to T2, the model revealed a significant positive cross-relation from children’s problem behavior to parental strain, *β*s = 0.18, *p* < 0.001, and a small negative cross-relation from parental strain to children’s problem behavior, *β*s = − 0.06, *p*s < 0.05. That means the higher children’s problem behavior at T1, the higher parental strain at T2. And the higher parental strain at T1, the lower children’s problem behavior at T2. Importantly, the cross-relation between children’s problem behavior and parental strain was moderated by the negative aspect of parent–child relationship quality, *β* = 0.06, SE = 0.04, *p* = 0.038: Children’s problem behavior at T1 was positively related to parental strain at T2, particularly if the negative aspect of parent–child relationship quality at T1 was high. A follow-up simple slope analysis on the respective multiple regression including the interaction term revealed a significant relation between child problem behavior at T1 and parental strain at T2 for low (-1 *SD*; *b* = 0.07, *p* = 0.019), medium (*M*; *b* = 0.11, *p* < 0.001), and high (+ 1 *SD*; *b* = 0.16, *p* < 0.001) levels of negative aspects of relationship quality. That is, the relation between child problem behavior at T1 and parental strain at T2 was significantly positive for all levels of relationship quality but differed in its strength (see Fig. 3). All other interactions in the CLPMs were non-significant, *β*s < 0.04, *p*s > 0.082.

In order to shed light on the impact of external regulations (strict lockdown versus looser regulations) on relations between parental strain and child variables, we exploratively examined whether these relations differed significantly between the two measurement points. For that purpose, we *z*-transformed the zero-order Pearson correlations and examined whether their 95% CI overlap. If this is not the case, the two correlations differ significantly. For all aspects of children’s well-being and problem behavior, relations with parental strain were significantly stronger at T1 than T2. Emotional well-being and parental strain were more strongly negatively correlated at T1, *r*(888) =  − 0.54, *p* < 0.001, than T2, *r*(875) =  − 0.22, *p* < 0.001. Likewise, family-related well-being, T1: *r*(888) =  − 0.41, *p* < 0.001; T2: *r*(875) =  − 0.22, *p* < 0.001, were more strongly negatively related to parental strain at T1 than T2. Children’s problem behavior and parental strain were more strongly positively correlated at T1, *r*(888) = 0.51, *p* < 0.001, than T2, *r*(873) = 0.37, *p* < 0.001. Parental strain was thus more strongly associated with child outcomes during the time of the strict lockdown compared to afterwards.

Taken together, parental strain was related to children’s well-being and problem behavior within measurement points. Results of the CLPM highlight longitudinal relations from child variables to parental strain rather than vice versa. Importantly, the findings show that the longitudinal relations between child and parent variables depend on the relationship quality.

### True Intraindividual Change Models Across T1 and T2 (*N* = 890)

TIC models are particularly suitable to examine factors that drive developmental change, because they allow to investigate whether change in one variable over two measurement points predicts intraindividual change in another variable. We computed separate TIC models for children’s emotional well-being (emotions, moods, life satisfaction), children’s family-related well-being and children’s problem behavior to investigate effects of parental strain on the respective child variable.

#### Parental Strain and Children’s Well-Being

The model addressing children’s *emotional wellbeing (emotions, moods, life satisfaction)* revealed an acceptable model fit, except for RMSEA, with χ^2^ (1, n = 890) = 14.55, *p* < 0.001, RMSEA = 0.12, SRMR = 0.03, CFI = 0.97. Parental strain at T1 positively predicted the change in children’s emotional well-being, *β* = 0.30, *SE* = 0.05, *p* < 0.001, *95% CI* [0.335, 0.529]. That means the higher parental strain at T1, the greater the increase in children’s emotional well-being across time. The change in parental strain from T1 to T2 negatively predicted the change in children’s emotional well-being, *β* = − 0.17, *SE* = 0.03, *p* < 0.001, *95% CI* [− 0.273, − 0.139]. The greater the decrease in parental strain, the greater the increase in children’s emotional well-being.

The model addressing children’s *family-related well-being* revealed a good model fit, with χ^2^ (1, n = 890) = 3.17, *p* = 0.075, RMSEA = 0.05, SRMR = 0.01, CFI = 0.99. Parental strain at T1 positively predicted the change in children’s family situation, *β* = 0.24, *SE* = 0.04, *p* < 0.001, *95% CI* [0.211, 0.369]. That means the higher parental strain at T1, the greater the increase in children’s family-related well-being across time. The change in parental strain negatively predicted the change in children’s family situation, *β* = − 0.15, *SE* = 0.03, *p* < 0.001, *95% CI* [− 0.197, − 0.098]. The greater the decrease in parental strain, the greater the increase in children’s family-related well-being.

#### Parental Strain and Children’s Problem Behavior

The model addressing children’s *problem behavior (emotional, conduct, hyperactivity)* revealed an acceptable model fit, except for RMSEA, with χ^2^ (1, n = 890) = 24.02, *p* < 0.001, RMSEA = 0.16, SRMR = 0.04, CFI = 0.98. Parental strain at T1 negatively predicted the change in children’s problem behavior, *β* = − 0.22, *SE* = 0.05, *p* < 0.001, *95% CI* [-0.396, -0.208]. That means the higher parental strain at T1, the lower the change in children’s problem behavior across time. The change in parental strain from T1 to T2 positively predicted the change in children’s problem behavior, *β* = 0.23, *SE* = 0.04, *p* < 0.001, *95% CI* [0.197, 0.342]. The more parental strain decreased, the more children’s problem behavior decreased.

**Fig. 4:**
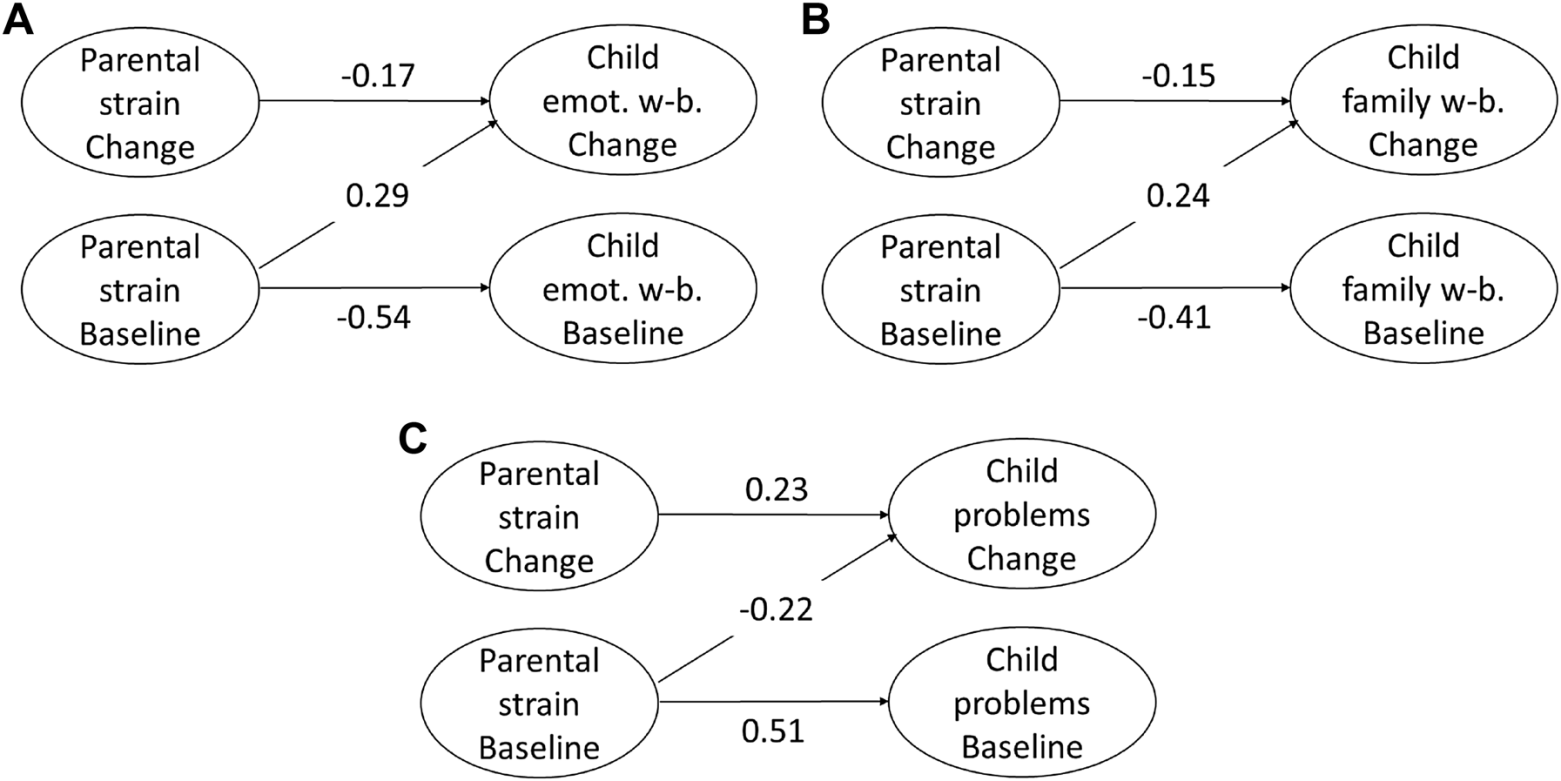
True intraindividual change models: Results for children’s emotional well-being (emotions, moods, life satisfaction) (**A**), for children’s family-related well-being (**B**), and for children’s problem behavior (**C**). Values indicate standardized path coefficients. All paths: p < .05

Taken together, the change in parental strain predicted the change in children’s emotional well-being, family-related well-being, and problem behavior. Results of the TIC models highlight the change of parental strain from T1 to T2 as a predictor of intraindividual change in children’s well-being and problem behavior across the same time (see Fig. 4).

## Discussion

The present study aimed at uncovering the developmental dynamics of child well-being and problem behavior during the COVID-19 pandemic. Most importantly, it is among the first to explore the temporal dynamics between parental strain and child well-being and problem behavior in a phase of quick and intense societal change. To this end, the current study employed a longitudinal developmental framework by assessing parents’ self-reports on a wide range of parent and child variables at two measurement points—one at the peak of the COVID-19 induced lockdown and one after restrictions had been majorly loosened. A key focus was on the impact of parental strain on child well-being and problem behavior, and the protective role of parent–child relationship quality.

Overall, results indicated that during the strict lockdown restrictions, children’s well-being and problem behavior were most strongly related to parental strain. As restrictions were loosened, parental stress as well as children’s problem behavior decreased while child emotional well-being increased. Interestingly, findings from CLPMs showed that longitudinal relations were more pronounced for child variables (well-being, problem behavior) predicting parental strain than the other way around with parent–child relationship quality as a moderator. Importantly, TIC models revealed the change of parental strain to be a predictor of the change in children’s well-being and problem behavior. This finding identifies the change in parental strain as a factor that drives develepomental change in children’s well-being and behavior. Overall, these results point to clear developmental changes in children’s and parents’ experiences of the pandemic and suggest important avenues for interventions.

Notably, our results are among the first to show the developmental trajectories of key child and parent indicators of well-being across the first phase of the COVID-19 pandemic. That is, there are tremendous changes from the peak of the lockdown restrictions to a situation about 10 weeks later where many restrictions had been loosened (e.g., partial reopening of educational facilities, reduced social distancing measures). Especially parental strain and children’s problem behavior have decreased over this period while child well-being increased. These findings highlight the impact of the COVID-19 related protocols on families and children.

Moreover, these findings pertain to developmental theorizing in a number of ways. First, they underscore notions of bioecological models pointing to the significance of especially the meso- and macrosocial context for child well-being [e.g., 7, 8]. That is, they provide unique insights into child and family functioning under drastic temporary changes of distal and consequently proximal factors. Thus, the disruption of macrosocial structures, which can only be tested in rare occasions like economic crises or pandemics, directly impacts on child well-being and family dynamics [see also 24–26]. Second, these results make a strong case for the external dependency of children in their well-being, coping, and behavior. Given that children’s cognitive, self-regulative, and emotional capacities changed only slightly over this time frame, the above effects can be considered as caused by factors external to the child. Consequently, our findings indicate the influence of external disruptions on children’s lives and point to the support children’s needs in successfully navigating changing social environments.

At times of the strict lockdown children’s well-being was strongly related to parental strain. This finding highlights the close interplay of child and caregiver psychological functioning. This is particularly pronounced at T1, which might stem from the intense contact at home and reduced external resources resulting from the general lockdown. In addition, a positive relationship quality and high parental self-efficacy emerged as protective factors, keeping child problem behavior low even though being faced with a challenging situation. This finding aligns with previous research reporting higher parental stress and parental worries to be a risk factor during the COVID-19 pandemic [e.g., 4, 52].

The cross-relations from child well-being and problem behavior during the lockdown to later parental strain outweighed cross-relations from parental strain to later child variables. That is, parental strain seems to be connected to previous child well-being to a much greater extent than the other way around. An explanation here could be that children’s situation-related well-being affects parental strain straightforwardly, particularly given a non-optimal relationship quality, because they completely depend on the family system in times of the lockdown. Parents, on the other hand, might have more resources or opportunities to regulate the expression of their strain. Situational parental strain might thus not affect child well-being in such a direct way.

The finding that parent–child relationship quality acted as a moderator concerning the effect of child variables (well-being and problem behavior) on parental strain is especially noteworthy. Both relationship dimensions (positive, negative) show a specific pattern: While a high level of positive aspects of parent–child relationship quality buffers the negative effect of child well-being on parental strain, a high level of negative aspects intensifies negative effects of child problem behavior on parental strain. This relates well to findings showing parent–child relationship quality to be an important moderator of the effects of parent–child acculturation gaps on child outcomes such as externalizing behavior [53]. It is also in line with findings showing that only for negative relationship quality, child maltreatment is related to lower levels of emotion regulation, which in turn predicts higher levels of internalizing and externalizing behavior [54]. Yet, relationship quality moderated the link from child well-being to parental strain, rather than the link vice versa that we expected. The finding suggests that also parents benefit from a positive relationship quality, as they might be able to better cope with children’s problems. Thus, the present study extends previous findings by showing that a positive parent–child relationship quality can be considered an important resilience factor, especially in challenging social environments. Regarding public policies, the present research suggests that the parent–child relationship quality could be a promising avenue for interventions during the pandemic. That is, interventions furthering positive aspects of the parent–child relationship might be particularly well suited to buffer against negative effects of future pandemic-related restrictions on both, the parent and the child side.

The TIC models provide unique insights into the factors predicting children’s situation-related adjustment to the COVID-19 pandemic in their well-being and problem behavior. That is, our results show that children’s changes in well-being and problem behavior are significantly predicted by both, parental strain at T1 as well as change in parental strain between T1 and T2. These results underscore theories relating caregiver availability and strain to child well-being [e.g., 17, 18]. In addition, it is a very interesting finding, as it suggests that there are two parental factors crucial for children’s adjustment to the COVID-19 pandemic [cf. 1]. First, parents’ initial response to the COVID-19 induced turmoil is of importance. This aspect relates to parents’ increased stress level as likely caused by the first days of the lockdown and the subsequent reorganizations of child care, work, and family routines. The greater this initial stress level, the greater the change in child emotional and family-related wellbeing and the smaller the change in child behavior problems. One possible interpretation here is that for initially highly stressed parents and subsequently initially highly stressed children, the possibility for the amelioration of children’s well-being and problem behavior was greater. Second, parents’ own adaptation to the COVID-19 pandemic policies as seen in their change of stress levels is a crucial factor. Specifically, decreases in parental strain predicted increases in child well-being and decreases in child problem behavior. It might be that decreased parental stress and increased parental well-being had positive effects on parenting practices [55] which in turn lead to improved child well-being and reduced problem behavior. Thus, one possible avenue for interventions during pandemic-related restrictions could be to especially provide social support for highly stressed parents (e.g., regarding the allocation of emergency child care places, financial support, online resources). Reducing stress could then in turn free up parental resources to cope with child-related and family-related issues. Given that our cross-lagged panel analyses showed child behavioral constructs as predictors of later parental adjustments, future research during the pandemic should also examine possible relations between child variables and changes therein as predictors of changes in parental adjustments.

Previous studies on the impact of the COVID-19 pandemic have identified a number of domains that could be potentially relevant to psychopathological symptoms and clinical interventions. That is, in the wake of the COVID-19 pandemic children experienced disturbed sleeping patterns and decreased sleep quality [6], symptoms of anxiety and depression were frequently reported in children and adolescents [e.g., 2, 13], and parental anxiety and depression were associated with higher parental stress and child abuse potential [4]. The present findings relate well to these studies and extend them by showing longitudinal risk factors that might pave the way for maladaptive, psychopathological developments in children while also pointing to protective factors fostering adaptive outcomes. In terms of risk factors, our results suggest that increasing parental stress and a low parent child relationship quality clearly bear on children’s emotional challenges, hyperactivity-related problems and general problem behavior. There seem to be potential pathways to psychopathological conditions related to emotional dysregulation, poor self-regulation, hyperactivity, and decreased overall well-being. In terms of protective factors, the coping capacities and stress levels of caregivers seem to be the most promising aspects contributing to adaptive development. This suggests that clinical interventions during the pandemic should especially focus on caregivers’ well-being and coping abilities to promote a stable microstructural environment and prevent psychopathological developments.

While these findings provide unique insights, some limitations have to be noted. First, due to the limited accessibility of families during the lockdown and the aim of a high sample size, we relied on parental (self-)report measures. It is possible that parents’ reports about their children’s situation might be biased. Second, while the current study relied on materials based on validated scales, a few adaptations were necessary in order to fit the extraordinary situation. Third, the retention rate from T1 to T2 was rather low. At T2, when restrictions had been loosened, many parents might have returned to activities outside of the household, hence finding less time to take part in the study. Third, the present study relied on parent-report data due to the initial lockdown restrictions. Future work should additionally include child-based measures. Fourth, while the adaptation of scales (e.g., SDQ) was warrented due to the special circumstances during the lockdown period, it also prevents comparisons with norm values.

Taken together, our study emphasizes the complex interaction of caregivers and children for families’ adjustment to the quickly changing COVID-19 situation. The findings point to parental behaviors as possible starting points for COVID-19 related interventions.

## Summary

The tremendous sweep of COVID-19 across the globe has uprooted human life. As our knowledge about the effects of the COVID-19 pandemic is still very limited, scientists have identified children and families as populations possibly particularly vulnerable. While parents are faced with new work settings and increased childcare demands, children might be in great need for emotional support, caregiver availability, and reliable relationships to caregivers. The present study employed a longitudinal developmental framework to provide unique insights into the relations between parental strain, child well-being, and child problem behavior while also examining the moderating role of parent–child relationship quality. At two measurement points (the first at the peak of the lockdown restrictions (*N* = 2921), the second after restrictions had been majorly loosened (*N* = 890)), parents reported their stress level, the parent–child relationship quality, and their child’s well-being and problem behavior. Results showed that at the peak of the restrictions, parental strain was negatively related to child well-being and positively related to child problem behavior. Between measurement points, parental stress and child problem behaviors decreased while child well-being increased. Cross-lagged panel analyses revealed longitudinal effects of child well-being and problem behavior at T1 on parental strain at T2 and the moderating role of the parent–child relationship quality, which acts as a protective factor. Finally, true intraindividual change models showed that decreases in parental strain between measurement points predicted increases in child well-being as well as decreases in child problem behavior. Taken together, our results indicate the strong impact of the COVID-19 pandemic on children and families. In addition, our results highlight the complex interaction between parental well-being and child well-being during the quickly changing COVID-19 environment. Thus, the present research points parental stress coping and child emotional adjustment as promising avenues for policy makers in their efforts to ensure child and family well-being throughout the pandemic.

## References

[1] Prime H, Wade M, Browne DT (2020). Risk and resilience in family well-being during the COVID-19 pandemic. Am Psychol.

[2] Xie X, Xue Q, Zhou Y, Zhu K, Liu Q, Zhang J, Song R (2020). Mental health status among children in home confinement during the coronavirus disease 2019 outbreak in Hubei Province, China. JAMA Pediatr.

[3] Wade M, Prime H, Browne DT (2020). Why we need longitudinal mental health research with children and youth during (and after) the COVID-19 pandemic. Psychiatry Res.

[4] Brown SM, Doom JR, Lechuga-Peña S, Watamura SE, Koppels T (2020). Stress and parenting during the global COVID-19 pandemic. Child Abuse Negl.

[5] Chin M, Sung M, Son S, Yoo J, Lee J, Chang YE (2020). Changes in family life and relationships during the COVID-19 pandemic and their associations with perceived stress. Fam Environ Res.

[6] Dellagiulia A, Lionetti F, Fasolo M, Verderame C, Sperati A, Alessandri G (2020). Early impact of COVID-19 lockdown on children’s sleep: a 4-week longitudinal study. J Clin Sleep Med.

[7] Bronfenbrenner U, Morris P (2006) The bioecological model of human development. In: Lerner RM, Damon W (eds) Handbook of child psychology: vol. 1. Theoretical models of human development, 6th edn. Wiley, pp 793–828

[8] Browne DT, Plamondon A, Prime H, Puente-Duran S, Wade M (2015). Cumulative risk and developmental health: an argument for the importance of a family-wide science. Wiley Interdiscip Rev Cogn Sci.

[9] Barnard CP (1994). Resiliency: a shift in our perception?. Am J Fam Ther.

[10] Feldman DH (2012) Cognitive development in childhood. In Weiner IB (ed) Handbook of psychology: vol 6. Developmental psychology, 2nd edn. Wiley, New York, pp 197–214. 10.1002/9781118133880.hop206008

[11] Best JR, Miller PH (2010). A developmental perspective on executive function. Child Dev.

[12] Malti T, Noam GG (2016). Social-emotional development: from theory to practice. Eur J Dev Psychol.

[13] Marques de Miranda D, da Silva AB, Sena Oliveira AC, Simoes-e-Silva AC (2020). How is COVID-19 pandemic impacting mental health of children and adolescents?. Int J Disaster Risk Reduct.

[14] Bowlby J (1969) Attachment and loss, 2nd edn. Basic Books, New York

[15] Calkins SD, Hill A, Gross JJ (2007). Caregiver influences on emerging emotion regulation. Handbook of emotion regulation.

[16] Thompson RA, Meyer S, Gross JJ (2007). Socialization of emotion regulation in the family. Handbook of emotion regulation.

[17] Julian MM, Lawler JM, Rosenblum KL (2017). Caregiver-child relationships in early childhood: interventions to promote well-being and reduce risk for psychopathology. Curr Behav Neurosci Rep.

[18] Sroufe LA (2005). Attachment and development: a prospective, longitudinal study from birth to adulthood. Attach Hum Dev.

[19] Thompson RA (2000). The legacy of early attachments. Child Dev.

[20] Brophy-Herb HE, Stansbury K, Bocknek E, Horodynski MA (2012). Modeling maternal emotion-related socialization behaviors in a low-income sample: relations with toddlers’ self-regulation. Early Child Res Q.

[21] Morelen D, Shaffer A, Suveg C (2016). Maternal emotion regulation: links to emotion parenting and child emotion regulation. J Fam Issues.

[22] Morris AS, Silk JS, Steinberg L, Myers SS, Robinson LR (2007). The role of the family context in the development of emotion regulation. Soc Dev.

[23] Paulus M, Licata M, Gniewosz B, Sodian B (2018). The impact of mother-child interaction quality and cognitive abilities on children’s self-concept and self-esteem. Cogn Dev.

[24] Brooks-Gunn J, Schneider W, Waldfogel J (2013). The Great Recession and the risk for child maltreatment. Child Abuse Negl.

[25] Schneider W, Waldfogel J, Brooks-Gunn J (2015). The great recession and behavior problems in 9-year old children. Dev Psychol.

[26] Schneider W, Waldfogel J, Brooks-Gunn J (2017). The Great Recession and risk for child abuse and neglect. Child Youth Serv Rev.

[27] Ashford J, Smit F, Van Lier PAC, Cuijpers P, Koot HM (2008). Early risk indicators of internalizing problems in late childhood: a 9-year longitudinal study. J Child Psychol Psychiatry.

[28] Masarik AS, Conger RD (2017). Stress and child development: a review of the Family Stress Model. Curr Opin Psychol.

[29] Mackler JS, Kelleher RT, Shanahan L, Calkins SD, Keane SP, O’Brien M (2015). Parenting stress, parental reactions, and externalizing behavior from ages 4 to 10. J Marriage Fam.

[30] Christner N, Essler S, Hazzam A, Paulus M (2021) Children’s psychological well-being and problem behavior during the COVID-19 pandemic: an online study during the lockdown period in Germany. PLoS ONE 10.1371/journal.pone.0253473 PMC8221463 34161376

[31] Walsh F (2015). Strengthening family resilience.

[32] Russell BS, Hutchison M, Tambling R, Tomkunas AJ, Horton AL (2020). Initial challenges of caregiving during COVID-19: caregiver burden, mental health, and the parent-child relationship. Child Psychiatry Hum Dev.

[33] Hazel NA, Oppenheimer CW, Technow JR, Young JF, Hankin BL (2014). Parent relationship quality buffers against the effect of peer stressors on depressive symptoms from middle childhood to adolescence. Dev Psychol.

[34] Miner JL, Clarke-Stewart KA (2008). Trajectories of externalizing behavior from age 2 to age 9: relations with gender, temperament, ethnicity, parenting, and rater. Dev Psychol.

[35] Papp LM, Cummings EM, Goeke-Morey MC (2005). Parental psychological distress, parent - child relationship qualities, and child adjustment: direct, mediating, and reciprocal pathways. Parent.

[36] Bandura A (1977). Self-efficacy: toward a unifying theory of behavioral change. Psychol Rev.

[37] Bogenschneider K, Small SA, Tsay JC (1997). Child, parent, and contextual influences on perceived parenting competence among parents of adolescents. J Marriage Fam.

[38] Hill NE, Bush KR (2001). Relationships between parenting environment and children’s mental health among African American and European American mothers and children. J Marriage Fam.

[39] Peterson L, Tremblay G, Ewigman B, Saldana L (2003). Multilevel selected primary prevention of child maltreatment. J Consult Clin Psychol.

[40] Jones TL, Prinz RJ (2005). Potential roles of parental self-efficacy in parent and child adjustment: a review. Clin Psychol Rev.

[41] Moshagen M, Erdfelder E (2016) SemPower. https://cran.r-project.org/web/packages/ordinal/ordinal.pdf

[42] Kline RB (2005) Principles and practice of structural equation modeling, 2nd edn. Guilford Press

[43] Ravens-Sieberer U (2006) The Kidscreen questionnaires: quality of life questionnaires for children and adolescents; handbook. Pabst Science Publishers

[44] Goodman R (1997). The strengths and difficulties questionnaire: a research note. J Child Psychol Psychiatry.

[45] Stone LL (2010). Psychometric properties of the parent and teacher versions of the Strengths and Difficulties Questionnaire for 4- to 12-year-olds: a review. Clin Child Fam Psychol Rev.

[46] Kliem S, Kessemeier Y, Heinrichs N, Döpfner M, Hahlweg K (2014). The parenting self-efficacy questionnaire (FSW). Diag.

[47] Furman W, Buhrmester D (1985). Children’s perceptions of the personal relationships in their social networks. Dev Psychol.

[48] Rosseel Y (2012) Lavaan. https://cran.r-project.org/web/packages/ordinal/ordinal.pdf

[49] Steyer R, Partchev I, Shanahan M, Little TD, Schnabel KU, Baumert J (2000). Modeling true intra-individual change in structural equation models: the case of poverty and children’s psychosocial adjustment. Modeling longitudinal and multiple-group data: practical issues, applied approaches, and specific examples.

[50] Graham JW (2009). Missing data analysis: making it work in the real world. Annu Rev Psychol.

[51] Meade AW, Johnson EC, Braddy PW (2008). Power and sensitivity of alternative fit indices in tests of measurement invariance. J Appl Psychol.

[52] Waller R, Powell T, Rodriguez Y, Corbett N, Perlstein S, White LK (2021). The impact of the COVID-19 pandemic on children’s conduct problems and callous-unemotional traits. Child Psychiatry Hum Dev.

[53] Schofield TJ, Parke RD, Kim Y, Coltrane S (2008). Bridging the acculturation gap: parent-child relationship quality as a moderator in Mexican American families. Dev Psychol.

[54] Alink LRA, Cicchetti D, Kim J, Rogosch FA (2009). Mediating and moderating processes in the relation between maltreatment and psychopathology: mother-child relationship quality and emotion regulation. J Abnorm Child Psychol.

[55] Abidin RR (1992). The determinants of parenting behavior. J Clin Child Psychol.

